# Dynamic changes of Ct values of N gene and ORF1ab genes and laboratory parameters in patients with COVID-19 caused by SARS-CoV-2 B.1, BA.2, and BA.5 variants and their correlation with clinical characteristics

**DOI:** 10.3389/fmicb.2025.1606306

**Published:** 2025-08-05

**Authors:** Wenjing Yang, Taoran Chen, Qi Zhou, Jiancheng Xu

**Affiliations:** ^1^Center of Infectious Diseases and Pathogen Biology, The First Hospital of Jilin University, Changchun, China; ^2^Department of Laboratory Medicine, The First Hospital of Jilin University, Changchun, China

**Keywords:** COVID-19, SARS-CoV-2 BA.2 variant, SARS-CoV-2 BA.5 variant, SARS-CoV-2 B.1 lineage, cycle threshold, reverse transcription-quantitative polymerase chain reaction

## Abstract

**Objective:**

To characterize the dynamic patterns of ORF1ab and N gene Ct values in oropharyngeal swabs from COVID-19 patients infected with different SARS-CoV-2 variants and assess their clinical and laboratory correlations.

**Methods:**

We conducted a retrospective cohort study of 259 COVID-19 patients hospitalized in Jilin Province between 2021–2023. Comparative analyses were performed on: (1) variant-specific Ct value trajectories for ORF1ab and N genes, (2) nucleic acid conversion times, and (3) longitudinal hematological and biochemical parameters.

**Results:**

B.1 variant exhibited the lowest median Ct values (ORF1ab: 31.37; N gene: 30.49) and longest median nucleic acid conversion time (18 days). BA.2 variant demonstrated the highest median Ct values (ORF1ab: 33.00; N gene: 32.00) and shortest conversion time (14 days). Disease progression correlated with: increased creatinine (CREA), neutrophil percentage (NE%), and coagulation markers (D-dimer). Decreased lymphocyte percentage (LY%).

**Conclusion:**

Significant inter-variant differences were observed in viral clearance kinetics (Ct values and conversion times) and organ dysfunction markers. These findings highlight variant-specific pathophysiological profiles, with B.1 showing prolonged viral shedding and Omicron subvariants (particularly BA.2) exhibiting faster clearance but distinct hematological perturbations.

## Introduction

SARS-CoV-2 has undergone genetic diversification since the COVID-19 pandemic caused by it, which has caused the introduction of novel variants (Alkhatib et al., 2021). The World Health Organization (WHO) has been regularly monitoring and evaluating the evolution of SARS-CoV-2 (Flisiak et al., 2023). Five variants of concern (VoC) have been detected and labeled with Greek letters to date: Alpha (B.1.1.7), Beta (B.1.351), Gamma (P.1), Delta (B.1.617), and Omicron (B.1.1.529). The B.1 lineage, which is regarded as the ancestral lineage and is less developed than the VoC above, may have originated during the COVID-19 outbreak in northern Italy in early 2020, as demonstrated (Gao et al., 2023). A novel SARS-CoV-2 variant (B.1.1.529) was discovered in South Africa from a patient sample on November 9, 2021, and the WHO named it the Omicron variant on November 26 (Colson et al., 2022). Due to a significant number of mutations (particularly in the spike and envelope proteins), studies have shown that Omicron variants exhibit increased transmissibility, high receptor binding affinity, and evasion of naturally occurring infection or vaccine-induced immunity (Sohn et al., 2022; Liu et al., 2025). This lineage consists of BA.1/21K, BA.2/21L, BA.3, BA.4/22A, BA.5/22B, BA.2.12.1/22C, and BA.2.75/22D; regarding the Omicron BA.5/22B variant, it was reported to have a growth advantage over the Omicron BA.1 and BA.2 variants (Colson et al., 2022).

Patients with COVID-19 typically present with systemic symptoms like fever and muscle pains or respiratory symptoms like cough (Long et al., 2022). The previous SARS-CoV-2 variants appear to affect primarily the upper respiratory tract and cause acute laryngitis without olfactory dysfunction, with clinical manifestations similar to those of epiglottitis in some patients, whereas the Omicron variant appears to affect primarily the lower respiratory tract and causes loss of smell and taste in many patients (Piersiala et al., 2022). In addition, patients with the Omicron variant show lower median ages and a higher proportion of milder and asymptomatic patients than those with the Delta and Beta variants (Yang et al., 2022).

Real-time reverse transcription polymerase chain reaction (RT-PCR) has been frequently used to identify SARS-CoV-2 nucleic acids in respiratory swabs from patients. When the detection value of the fluorescent signal is above the cycling threshold (Ct), the earlier the cycle, the higher the concentration of the target gene in the sample. The Ct value is typically inversely proportional to the viral load (Aranha et al., 2021), and the Ct value is frequently utilized as a distinct metric of viral load in the research of new coronaviruses (Miranda et al., 2021).

In Jilin Province, China, COVID-19 patients appeared one after the other from 2021 to 2023. The three SARS-CoV-2 variants that predominated during that time all belonged to different genotypes, as revealed by whole genome sequencing and genomics analysis. The BA.2 variant prevalent in 2022 and the BA.5 variant prevalent in 2023 were both members of the Omicron lineage (Colson et al., 2022), while the B.1 variant prevalent in 2021 belonged to the ancestral lineage (Gao et al., 2023). In this study, the Ct values of the ORF1ab gene and the N gene of SARS-CoV-2 in oropharyngeal swabs of patients with COVID-19, the negative conversion time of nucleic acid, clinical performance of patients with COVID-19, and blood test levels were compared and analyzed in order to investigate the characteristics of molecular biological tests and other laboratory tests for different genotypes of SARS-CoV-2, as well as the differences in clinical performance, and to provide a reference for the diagnosis and treatment of COVID-19.

## Materials and methods

### Participants and clinical samples

A total of 259 patients were included in this study, including 110 patients with B.1 variant infection admitted to Changchun Infectious Disease Hospital from January to February 2021, 82 patients with BA.2 variant infection admitted to Department II of Jilin University Hospital from April to May 2022, and 67 patients with BA.5 variant infection admitted to Mehekou City Hospital from December 2022 to February 2023. The demographic data, clinical and laboratory parameters, and clinical classification were obtained through the hospital’s electronic medical record and analyzed by a group of skilled doctors.

In our study, clinical data included patient demographics (sex, age); “combined underlying disease” refers to pre-existing chronic conditions in patients, such as hypertension, diabetes, cardiovascular disease, pulmonary disease, renal disease, liver disease, neurological disease, and prior history of malignancy; signs and symptoms (fever, cough, chest tightness, weakness, palpitations, dyspnea, shortness of breath after activity, dizziness and headache, nausea and vomiting, nausea and diarrhea, asymptomatic), and duration of hospitalization.

Laboratory parameters included SARS-CoV-2 nucleic acid test results (ORF1ab gene and N gene Ct values) from the patient’s oropharyngeal swab and blood tests selected based on their established clinical relevance for COVID-19 severity assessment: (1) Renal function markers: creatinine (CREA, reference interval 41–73 10^9^/L): elevated levels correlate with acute kidney injury, a known complication of severe COVID-19 (Cheng et al., 2020). (2) Inflammatory and hematologic markers: white blood cells (WBC, 3.52–9.50 × 10^9^/L) and differential counts (NE% 40–75%, LY% 20–50%): lymphopenia and neutrophilia are hallmarks of SARS-CoV-2-induced cytokine storm (Terpos et al., 2020), and Platelet count (PLT, 125–350 × 10^9^/L): thrombocytopenia predicts poor outcomes in COVID-19 (Lippi et al., 2020). (3) Coagulation parameters: D-dimer (D-D, 0.00–0.50 mg/L): key predictor of thrombotic complications (Arachchillage and Laffan, 2020). APTT (28.0–42.0 s) and PT (11.0–15.0 s): coagulopathy indicators associated with disease severity (Levi et al., 2020). (4) Nutritional marker: Albumin (ALB, 40–55 × 10^9^/L): hypoalbuminemia reflects systemic inflammation and predicts mortality (Huang et al., 2020).

The disease is divided into four categories in clinical practice: mild, moderate, severe, and critical. Mild cases are those with mild clinical symptoms and no imaging signs of pneumonia; moderate cases are those with fever and respiratory symptoms and imaging signs of pneumonia; and severe cases are those with one of the following criteria: (a) dyspnea with respiratory rate ≥ 30/min; (b) pulse oximetry at rest ≤93%; (c) oxygenation index (arterial partial pressure of oxygen/inhalation oxygen fraction, PaO_2_/FiO_2_) ≤300 mmHg; (d) progressive clinical symptoms with significant progression of lesions >50% on lung imaging within 24–48 h; critical cases are those that meet one of the following criteria: (a) respiratory failure requiring mechanical ventilation; (b) shock; (c) combined with other organ failure requiring ICU monitoring and treatment. All diagnostic and clinical classifications of COVID-19 above are based on the “Diagnosis and Treatment Protocol for Novel Coronavirus Pneumonia (8th Trial Version)” published by the National Health Commission of the People’s Republic of China (Wei, 2020).

### Methods

In this study, pharyngeal swabs from patients with COVID-19 were collected for routine COVID-19 diagnosis, and the inclusion criterion was a Ct value of <38 for nucleic acid testing in patients in 2021–2022, and <35 for nucleic acid testing in 2023. The negative criteria for nucleic acid testing in 2021–2022 were established as two consecutive nucleic acid tests for the ORF1ab gene and the N gene with a Ct value of ≥38 (fluorescence quantitative PCR method, two sampling intervals of at least 24 h); the negative criteria for nucleic acid testing in 2023 were established as two consecutive nucleic acid tests for the ORF1ab gene and the N gene with a Ct value of ≥35 (fluorescence quantitative PCR method, two sampling intervals of at least 24 h). When patients were admitted to the hospital, blood tests were conducted every 3 days for mild patients and as often as the dynamics of the disease permitted until the patients were discharged for severe patients. Four clinical classification were present in the cases included in this study, but there were not enough patients with the moderate or critical types to form separate groups. As a result, the text has referred to the combination of mild and moderate patients as “mild” and the combination of severe and critical patients as “severe.”

### Quality control

Laboratory parameters included ORF1ab gene and N gene Ct value testing for SARS-CoV-2 in patient oropharyngeal swabs and blood tests. Oropharyngeal swab sample collection and RT-qPCR assays were performed by trained medical personnel according to standardized procedures and traceability of sample results. “Guidelines for the Implementation of Regional 2019-nCoV Nucleic Acid Detection (2nd Edition)” and “Working Manual of Novel Coronavirus Nucleic Acid Detection for Medical Institutions (second trial Edition)” were strictly followed during the sample collection procedure. RNA extraction was performed using a nucleic acid extraction (NAE) system and accompanying NAE or purification reagents, and the operating procedures strictly followed the manufacturer’s protocol. Nucleic acid amplification was performed on a RT-qPCR detection system. The PCR reaction system configuration, reaction parameters, and program settings were set according to the kit’s instructions, respectively. Each run was subjected to quality control, which included three weakly positive and one weakly negative controls to detect false positive and false negative results. At the three hospitals, the blood testing equipments were put through stringent quality control testing. The Jilin Clinical Laboratory Center accredited all three of the study’s participating labs for inter-laboratory quality evaluation and proficiency. The Health Commission of Jilin Province provided standardized training to all of the doctors, technicians, and nurses involved in this study. For common biochemical analytes and blood cell analysis in Chinese adults, reference interval standards have been issued by the National Health Commission of the People’s Republic of China. Reference intervals that met the requirements were used by the three hospitals. There was no influence of different instruments on the test results.

### Statistical analysis

All statistical analyses were performed using IBM SPSS Statistics version 22 (IBM Corporation, Armonk, NY, United States). Data distribution normality was assessed using the Shapiro–Wilk test. Continuous variables including Ct values, nucleic acid conversion time, and hematological parameters were analyzed as follows: for normally distributed data: group comparisons were conducted using unpaired Student’s *t*-tests, results expressed as mean ± standard deviation. For non-normally distributed data: Mann–Whitney *U* tests were employed, results presented as median (interquartile range, IQR). Categorical variables (demographic characteristics and clinical manifestations) were expressed as frequencies (percentages) and compared using: *χ*^2^ tests for expected cell counts >5; Fisher’s exact tests for expected cell counts ≤5. Correlation analyses were performed using: Pearson’s correlation for normally distributed continuous variables; Spearman’s rank correlation for non-parametric data; binary logistic regression for dichotomous outcomes. All statistical tests were two-tailed, with *p* < 0.05 considered statistically significant. Graphical representations were generated using GraphPad Prism 8 (GraphPad Software, San Diego, CA) and OriginPro 2022 (OriginLab Corporation, Northampton, MA).

### Ethics

The data set was entirely anonymous and did not include any personally identifiable health information that might be used to violate the subjects’ rights or interests. The study was carried out in accordance with the approved guidelines and was approved by the ethics committees of the hospitals: Ethics Committee of Changchun Infectious Diseases Hospital (No. 2020-001) and First Hospital of Jilin University (No. K2022028). The Ethics Committee of the First Hospital of Jilin University (No. K2022028) and the Ethics Committee of Changchun Infectious Diseases Hospital (No. 2020-001) waived written informed consent for this study for it was a retrospective study.

## Results

### Baseline characteristics of study population

Table 1 presents the demographic and clinical characteristics across the three SARS-CoV-2 variants. Key findings include: (1) Demographic distribution: No significant sex differences were observed among variants (*p* > 0.05). Significant age variations emerged: B.1 patients were younger (median: 56 years) than BA.2 (65 years) and BA.5 (72 years) cohorts (*p* < 0.05). (2) Comorbidity profiles: The prevalence of underlying diseases differed substantially (*p* < 0.05): B.1: 46.36% vs. BA.2: 67.07% vs. BA.5: 76.12%. (3) Disease severity: BA.5 variant demonstrated the highest proportion of severe cases (50.75%), contrasting with BA.2 (0%) and B.1 (8.18%) (*p* < 0.05). (4) Vaccination status: All B.1 variant cases were unvaccinated (100%). Comparable vaccination rates between BA.2 (45.12%) and BA.5 (34.33%) variants (*p* > 0.05).

**Table 1:** Comparison of basic characteristics of three variants.

Groups		B.1	BA.2	BA.5
Total		110	82	67
Sex (*n*, %)	Male	46 (41.82)	42 (51.22)	29 (43.28)
	Female	64 (58.18)	40 (48.78)	38 (56.72)
Median age		56^a^^,^^b^	65	72
Age (*n*, %)	<60	62 (56.36)^a^^,^^b^	31 (37.80)	18 (26.87)
	≥60	48 (43.64)^a^^,^^b^	51 (62.20)	49 (73.13)
Clinical classifications (*n*, %)	Mild	101 (91.82)^b^	82 (100)	33 (49.25)
	Severe	9 (8.18)^b^	0	34 (50.75)
Underlying diseases (*n*, %)	Yes	51 (46.36)^a^^,^^b^	55 (67.07)	51 (76.12)
	No	59 (53.64)^a^^,^^b^	27 (32.93)	16 (23.88)
Vaccination (*n*, %)	Yes	0	37 (45.12)	23 (34.33)
	No	110 (100)	36 (43.90)	17 (25.37)

a Indicates that there is a significant difference in data distribution between patients with B.1 and BA.2 variants.

b Indicates that there is a significant difference in data distribution between patients with B.1 and BA.5 variants; patients with BA.2 and BA.5 variants in all grouped data constituted differences that were not statistically significant.

### Viral load dynamics across variants

**Figure 1:**
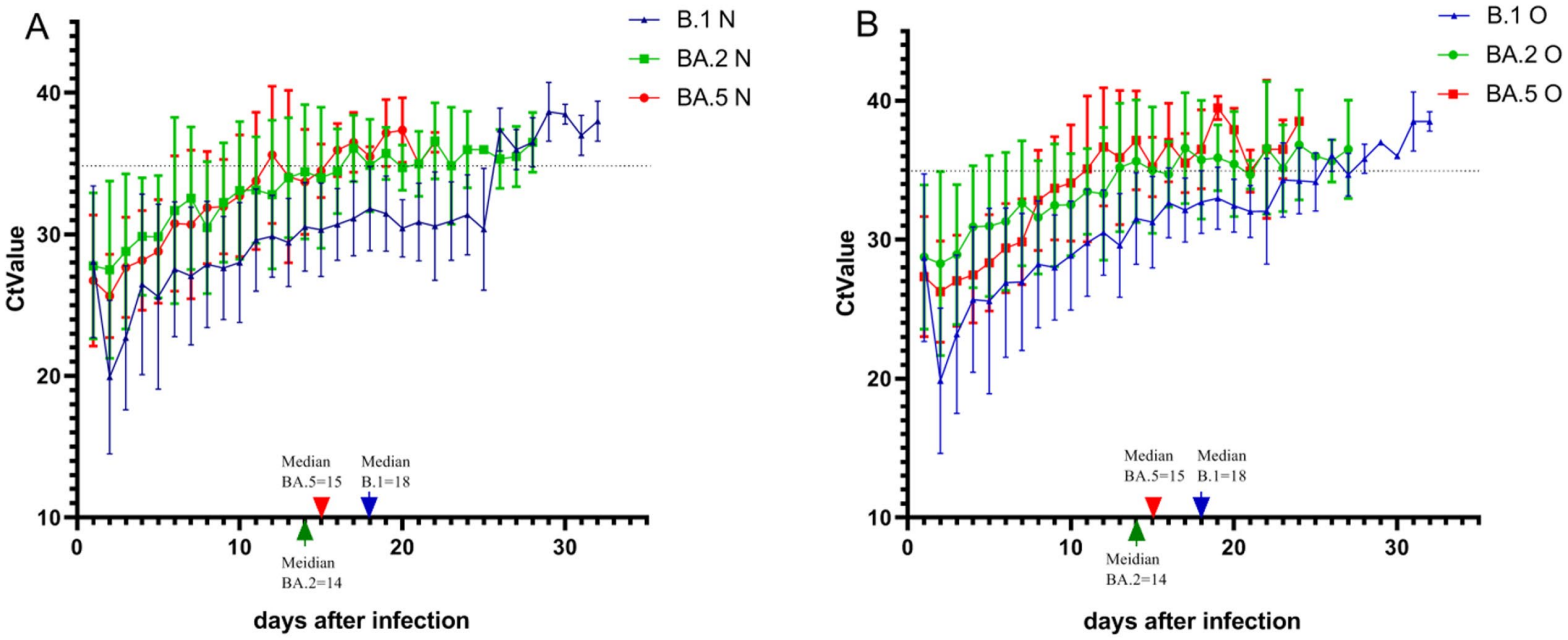
Temporal dynamics of ORF1ab and N gene cycle threshold values across three SARS-CoV-2 variant infections. Using the first positive nucleic acid test as the first day of the disease course, the distribution and trend of Ct values of the ORF1ab gene and the N gene in oropharyngeal swabs of patients with the three variants during the disease course were recorded. **(A)** Depicts the trend of Ct value change for the N gene. **(B)** Depicts the trend of Ct value change for the ORF1ab gene. The green arrow on the marked line in the figure indicates the median negative nucleic acid conversion time of 14 days for patients with BA.2 variant, the red arrow indicates the median negative nucleic acid conversion time of 15 days for patients with BA.5 variant, and the blue arrow indicates the median negative nucleic acid conversion time of 18 days for patients with B.1 variant. The Ct value corresponding to the dashed line in the figure is 35.

Our analysis revealed distinct patterns in Ct values (representing viral load) among the three variants (Table 2 and Figure 1): (1) Inter-variant comparisons: B.1 variant demonstrated significantly lower median Ct values (higher viral loads) for both ORF1ab (median: 31.37) and N genes (median: 30.49) compared to BA.2 (ORF1ab: 33.00; N: 32.00; both *p* < 0.05). The N gene consistently showed lower Ct values than ORF1ab across all variants. (2) Clinical severity association: B.1 severe cases exhibited significantly lower viral loads than mild cases (ORF1ab ΔCt = 2.58; N gene ΔCt = 2.22, both *p* < 0.05). (3) Omicron subvariants (BA.2 vs. BA.5): BA.2 showed higher Ct values (lower viral loads) than BA.5 overall (ORF1ab: 33.00 vs. 32.38; N: 32.00 vs. 31.54; both *p* < 0.05), this pattern persisted in vaccinated subgroups (ORF1ab: 32.00 vs. 30.96; N: 32.00 vs. 30.23, both *p* < 0.05). Gender differences were observed in BA.2 (Male vs. Female: ORF1ab 33.00 vs. 32.00; N 33.00 vs. 31.00, both *p* < 0.05). (4) Vaccination effects: Paradoxically, BA.5 vaccinated patients showed lower Ct values than unvaccinated (ORF1ab: 30.96 vs. 34.66; N: 30.23 vs. 33.34, both *p* < 0.05). (5) Longitudinal patterns: All variants showed progressive Ct value increases (viral load decline) over time (Figure 1).

**Table 2:** Comparison of Ct values of OFR1ab gene and N gene among patients with three variants.

		B.1		BA.2		BA.5	
		O	N	O	N	O	N
Median Ct value		31.37 (28.31, 33.44)^a^^,^^d^	30.49 (27.62, 32.54)^a^	33.00 (29.00, 36.00)^c^	32.00 (28.00, 35.00)^c^	32.38 (27.29, 34.70)	31.54 (26.35, 34.13)
Sex	Male	31.21 (28.17, 33.46)^a^^,^^b^^,^^d^	30.55 (27.87, 32.56)^a^	33.00 (29.00, 36.00)^e^	33.00 (29.00, 35.00)^c^^,^^e^	32.62 (27.43, 34.97)	32.00 (26.24, 34.23)
	Female	31.46 (28.36, 33.43)^a^^,^^d^	30.49 (27.51, 32.51)^a^	32.00 (28.00, 35.00)^e^	31.00 (27.00, 35.00)^e^	32.00 (27.19, 34.50)	30.93 (36.39, 34.12)
Age	<60	31.41 (28.68, 33.44)^a^^,^^d^	30.38 (27.79, 32.43)^a^	33.00 (29.00, 36.00)	32.00 (27.75, 35.00)	32.88 (26.87, 34.87)	32.00 (25.74, 34.12)
	≥60	31.29 (27.80, 31.29)^a^^,^^d^	30.59 (27.38, 32.73)^a^	32.00 (29.00, 35.00)	32.00 (28.00, 35.00)^c^	32.20 (27.65, 34.70)	31.15 (27.18, 34.18)
Clinical classifications	Mild	31.49 (28.61, 33.45)^a^^,^^d^^,^^e^	30.55 (27.80, 32.59)^a^^,^^e^	33.00 (29.00, 36.00)	32.00 (28.00, 35.00)	32.50 (26.69, 35.01)	32.04 (25.85, 34.56)
	Severe	28.91 (25.36, 31.81)^b^^,^^e^	28.33 (24.69, 31.63)^b^^,^^e^	—	—	31.99 (27.54, 34.40)	31.15 (27.39, 33.92)
Underlying diseases	Yes	31.16 (27.63, 33.42)^a^^,^^d^	30.43 (26.92, 32.40)^a^	32.00 (29.00, 35.00)^c^	32.00 (28.00, 35.00)^c^	32.07 (26.48, 34.96)	31.04 (25.48, 34.38)
	No	31.61 (28.78, 33.45)^a^^,^^d^	30.57 (28.12, 32.62)^a^^,^^b^	33.00 (29.00, 36.00)	33.00 (28.00, 36.00)	32.88 (31.06, 33.65)	32.40 (29.97, 33.49)
Vaccination	Yes	—	—	32.00 (29.00, 36.00)^c^	32.00 (28.00, 35.00)^c^	30.96 (25.41, 33.66)^e^	30.23 (23.82, 33.92)^e^
	No	31.37 (28.31, 33.44)^a^^,^^b^^,^^d^	30.49 (27.62, 32.54)^a^^,^^b^	32.00 (28.00, 36.00)	32.00 (27.00, 35.00)	34.66 (27.71, 35.11)^e^	33.34 (24.68, 37.39)^e^

a Indicates that difference between patients with B.1 and BA.2 has statistical significance.

b Indicates that difference between patients with B.1 and BA.5 has statistical significance.

c Indicates that difference between patients with BA.2 and BA.5 has statistical significance.

d Indicates that the OFR1ab gene and the N gene of patients has statistical significance.

e Indicates that OFR1ab or N genes in the gender, age, clinical classification, whether the merger basic diseases, whether vaccination interclass difference has statistically significant.

### Comparison of the negative nucleic acid conversion time of patients with the three variants

**Figure 2:**
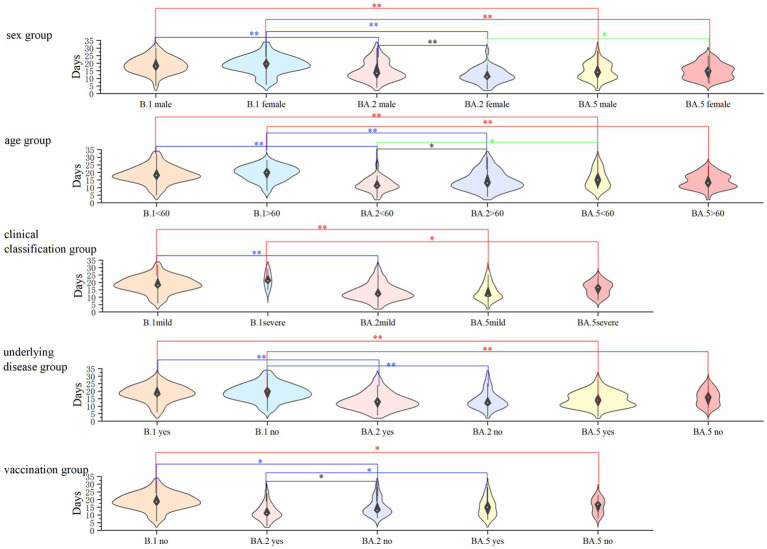
Comparison of nucleic acid conversion time across demographic and clinical subgroups in three SARS-CoV-2 variants. The graph describes the differences in the negative nucleic acid conversion time of patients with the three variants in the gender, age, underlying disease, clinical typing, and vaccination groups, where none of the patients with the B.1 variant were vaccinated and no patients with severe disease were infected with the BA.2 variant, so they are not indicated in the graph. ^*^Indicates *p* < 0.01 and indicates *p* < 0.05, and the differences were considered statistically significant.

Results of viral clearance kinetics analysis revealed significant differences among variants (Figure 1). The median time to negative nucleic acid conversion was longest in B.1 variant patients (18 days), followed by BA.5 (15 days) and BA.2 variants (14 days), with B.1 demonstrating significantly prolonged clearance compared to both Omicron subvariants in all stratified analyses (*p* < 0.01). Multivariate analysis showed that: for B.1 and BA.5 variants: no significant associations were observed between nucleic acid conversion time and sex, age, disease severity, comorbidities, or vaccination status (all *p* > 0.05). For BA.2 variant: advanced age correlated with prolonged viral clearance (*p* < 0.05). Unvaccinated status was associated with longer conversion time compared to vaccinated individuals (*p* < 0.05) (Figure 2). Notably, no significant inter-group differences were observed between BA.2 and BA.5 variants in other demographic or clinical subgroups (all *p* > 0.05). These findings suggest variant-specific patterns of viral persistence, with BA.2 showing unique age- and vaccine-dependent clearance dynamics.

### Comparison of the clinical performance of the three variants

Clinical symptom profiles differed significantly among the three variants (Table 3). While fever and cough represented the most prevalent symptoms across all variants, their clinical manifestations exhibited distinct patterns: (1) Asymptomatic presentation: The B.1 variant demonstrated the highest proportion of asymptomatic cases (*n* = 91, 82.73%), significantly exceeding BA.2 (*n* = 14, 17.07%) and BA.5 (*n* = 3, 4.48%) variants (*p* < 0.001). (2) Symptom spectrum: BA.2 and BA.5 variants were associated with broader symptom profiles, including: cardiorespiratory symptoms: chest distress (B.1: 1.82% vs. BA.2: 13.41% and BA.5: 35.82%, *p* < 0.05) and dyspnea (B.1: 0.91% vs. BA.2: 13.41% and BA.5: 16.42%, *p* < 0.05); Systemic manifestations: weakness (B.1:1.82% vs. BA.2: 20.73% and BA.5: 31.34%, *p* < 0.05) and myalgia (B.1: 0.91% vs. BA.2: 13.41% and BA.5: 16.42%, *p* < 0.05); Neurological symptoms: headache (B.1: 0 vs. BA.2: 12.20% and BA.5: 19.40%, *p* < 0.05). (3) Variant-specific features: BA.5 variant patients showed significantly higher rates of: shortness of breath after activity (BA.5: 26.87% vs. BA.2: 2.44%, *p* < 0.05); gastrointestinal symptoms (nausea/vomiting): (BA.5: 16.42% vs. BA.2: 2.44%, *p* < 0.05). These findings suggest evolutionary changes in viral tropism and pathogenicity across variants, with Omicron sublineages (particularly BA.5) exhibiting enhanced respiratory and systemic symptomatology.

**Table 3:** Comparison of clinical symptoms of patients with three variants.

	B.1	BA.2	BA.5
Asymptomatic	91 (82.73)^a^^,^^c^	14 (17.07)^b^	3 (4.48)
Fever	11 (10.00)^a^^,^^c^	17 (20.73)^b^	49 (73.13)
Cough	12 (10.91)^a^^,^^c^	41 (50.00)^b^	55 (82.09)
Palpitation	3 (2.73)^a^^,^^c^	7 (8.54)	13 (19.40)
Chest distress	2 (1.82)^a^^,^^c^	11 (13.41)^b^	24 (35.82)
Weakness	2 (1.82)^a^^,^^c^	17 (20.73)	21 (31.34)
Myalgia	1 (0.91)^a^^,^^c^	11 (13.41)	11 (16.42)
Headache	0	10 (12.20)	13 (19.40)
Dyspnea	1 (0.91)^a^^,^^c^	11 (13.41)	11 (16.42)
Diarrhea	2 (1.82)^a^	7 (8.54)	1 (1.49)
Shortness of breath after activity	0	2 (2.44)^b^	18 (26.87)
Nausea and vomiting	1 (0.91)^a^^,^^c^	2 (2.44)^b^	11 (16.42)

a Indicates that there is a significant difference in clinical symptoms between patients with B.1 and BA.2 variants.

b Indicates that there is a significant difference between patients with BA.2 and BA.5 variants.

c Indicates that there is a significant difference between patients with B.1 and BA.5 variants.

### Assessment of risk factors for clinical classification and clinical performance

**Figure 3:**
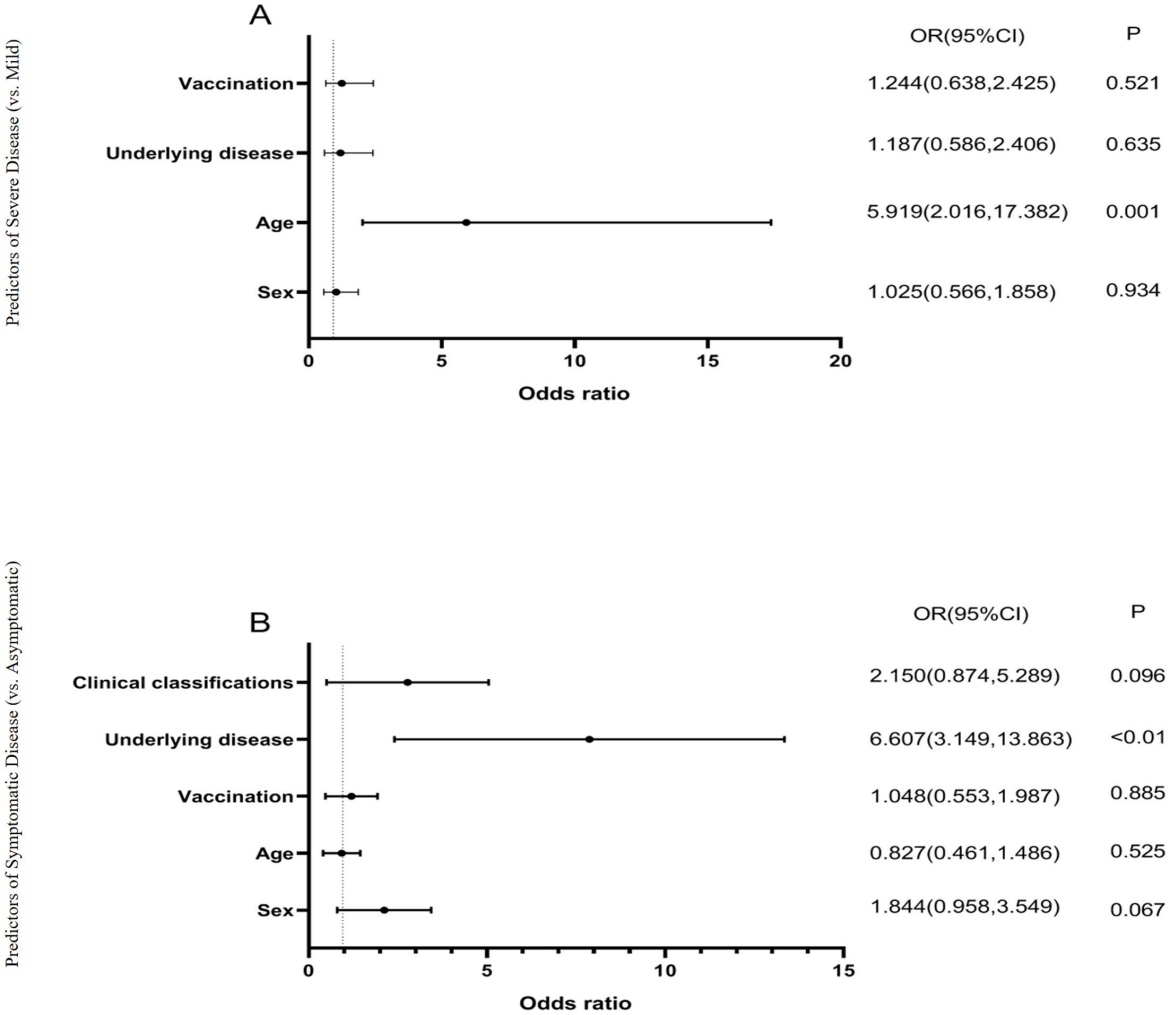
Logistic regression analysis of factors influencing COVID-19 clinical outcomes. **(A)** Predictors of severe disease (vs. mild), models the binary outcome of disease severity (severe vs. mild), analyzes four covariates: sex, age (≥60 vs. <60 years), underlying disease, and vaccination status. Odds ratios (OR) >1 indicate increased risk of severe disease, *p* < 0.01 considered statistically significant. **(B)** Predictors of symptomatic infection (vs. asymptomatic), models the binary outcome of symptom presentation (symptomatic vs. asymptomatic), analyzes five covariates: sex, age, vaccination status, underlying disease, and clinical classification. Odds ratios (OR) >1 indicate increased likelihood of symptomatic infection, *p* < 0.01 considered statistically significant. All models used logistic regression with maximum likelihood estimation. The horizontal lines represent 95% confidence intervals. Age ≥60 years was significantly associated with severe disease (OR = 5.919, *p* = 0.001), while underlying diseases were significantly associated with symptomatic infection (OR = 6.607, *p* < 0.01).

To assess risk factors associated with disease severity and clinical manifestations, we performed multivariate logistic regression analyses incorporating sex, age, underlying comorbidities, and vaccination status across all three variants. Key findings demonstrated: (1) Disease severity predictors (Figure 3A): advanced age (≥60 years) emerged as a significant independent risk factor for severe disease classification (adjusted OR = 5.92, 95% CI: 2.02–17.38; *p* < 0.001), after controlling for sex, comorbidities and vaccination status. (2) Symptomatic presentation predictors (Figure 3B): the presence of underlying comorbidities showed the strongest association with symptomatic disease (adjusted OR = 6.61, 95% CI: 3.15–13.86; *p* < 0.01), independent of age, sex or vaccination status. Notably, vaccination status did not demonstrate statistically significant associations with either outcome measure in our regression models (all *p* > 0.05). These findings highlight the differential impacts of demographic versus clinical factors on COVID-19 outcomes during the study period.

### Comparison of blood test levels for patients with the three variants

Patients infected with the BA.2 and BA.5 variants showed significantly higher serum creatinine (CREA), white blood cell count (WBC), and neutrophil percentage (NE%) compared to those infected with the B.1 variant (*p* < 0.01), while albumin (ALB) and lymphocyte percentage (LY%) were significantly lower than in B.1 variant patients.

**Figure 4:**
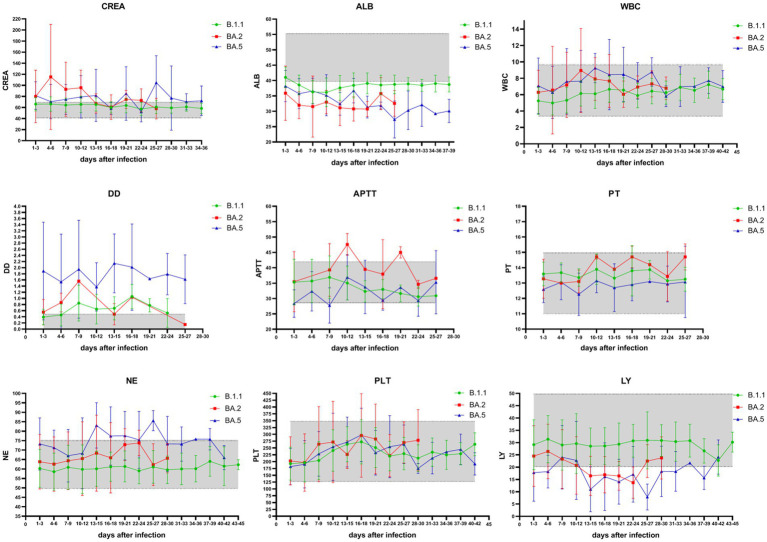
Temporal trends of key hematological and biochemical parameters in COVID-19 patients across SARS-CoV-2 variants. Green represents blood test results in patients with the B.1 variant, red represents blood test results in patients with the Omicron BA.2 variant, and blue represents blood test results in patients with the Omicron BA.5 variant. The shaded part of the graph represents the reference interval of blood test parameters. Measured parameters: CREA: creatinine (41–73 μmol/L); ALB: albumin (40–55 g/L); WBC: white blood cells (3.5–9.5 × 10⁹/L); NE%: neutrophil percentage (40–75%); LY%: lymphocyte percentage (20–50%); PLT: platelet count (125–350 × 10⁹/L); APTT: activated partial thromboplastin time (28–42 s); PT: prothrombin time (11–15 s); D-D: D-dimer (0.00–0.50 mg/L). During infection with the Omicron BA.2 and BA.5 variants, the measured values of CREA, ALB, D-D, and LY% in patients were mostly either above or below the reference ranges.

Among patients infected with the B.1 and BA.5 variants, as disease severity increased, LY% decreased significantly, whereas CREA, NE%, activated partial thromboplastin time (APTT), prothrombin time (PT), and D-dimer (D-D) increased significantly (Table 4). However, in patients infected with the B.1 variant, blood test parameters such as CREA, WBC, APTT, PT, NE%, platelet count (PLT), and LY% fluctuated within the reference ranges throughout the disease course. In contrast, in patients infected with the Omicron BA.2 and BA.5 variants, most measurements of CREA, ALB, D-D, and LY% were either above or below the reference ranges (Figure 4).

**Table 4:** Comparison of blood test results of patients with three variant strains.

	B.1		BA.2		BA.5	
Clinical classifications	Mild	Severe	Mild	Severe	Mild	Severe
Total (*n*, %)	101 (91.82)	9 (8.18)	82 (100)	0	33 (49.25)	34 (50.75)
CREA	62.00 (53.00, 72.00)^a^^,^^b^^,^^d^	65.00 (54.00, 81.75)^b^	74.50 (60.75, 89.25)	—	69.00 (53.00, 95.00)^d^	77.10 (63.00, 112.10)
ALB	38.60 (36.5, 40.9)^a^^,^^b^^,^^d^	31.45 (29.50, 34.50)^b^	33.3 (30.28, 37.35)	—	34.00 (30.00, 38.00)	34.00 (30.00, 37.00)
WBC	5.80 (4.60, 7.20)^a^^,^^b^^,^^d^	8.10 (6.45, 9.95)	6.26 (4.62, 8.03)^c^	—	6.97 (5.13, 9.46)	6.96 (5.25, 10.44)
NE%	60.50 (53.70, 66.20)^a^^,^^b^^,^^d^	78.70 (71.65, 86.45)^b^	65.70 (55.35, 75.19)^c^	—	74.30 (64.85, 83.15)^d^	81.60 (72.80, 91.00)
LY%	29.00 (24.00, 35.70)^a^^,^^b^^,^^d^	11.20 (7.70, 16.50)	22.15 (14.38, 29.80)^c^	—	17.20 (10.15, 24.25)^d^	10.60 (5.20, 17.70)
PLT	220.5 (176.0, 269.75)	220.0 (120.0, 296.5)^b^	209.0 (151.0, 273.0)	—	205.0 (168.0, 270.0)^d^	161.0 (124.0, 231.0)
APTT	33.00 (30.23, 37.20)^a^^,^^b^^,^^d^	34.25 (32.60, 39.50)^b^	36.30 (33.35, 42.65)^c^	—	29.70 (25.95, 35.20)^d^	33.05 (29.20, 36.03)
PT	13.40 (13.10, 13.88)^b^^,^^d^	15.90 (14.08, 17.63)^b^	13.40 (12.60, 14.20)^c^	—	12.70 (11.95, 13.50)^d^	13.80 (12.38, 15.20)
DD	0.47 (0.24, 0.83)^b^^,^^d^	2.02 (0.88, 3.76)	0.42 (0.26, 1.10)^c^	—	1.24 (0.82, 3.81)	1.71 (1.04, 3.01)

a Indicates that patients with B.1 variants and BA.2 variants blood test index difference has statistically significant.

b Indicates that patients with B.1 variants and BA.5 variants blood test index difference has statistically significant.

c Indicates that patients with BA.2 variants and BA.5 variants blood test index difference has statistically significant.

d Indicates that mild and severe patients blood test index difference has statistically significant.

**Figure 5:**
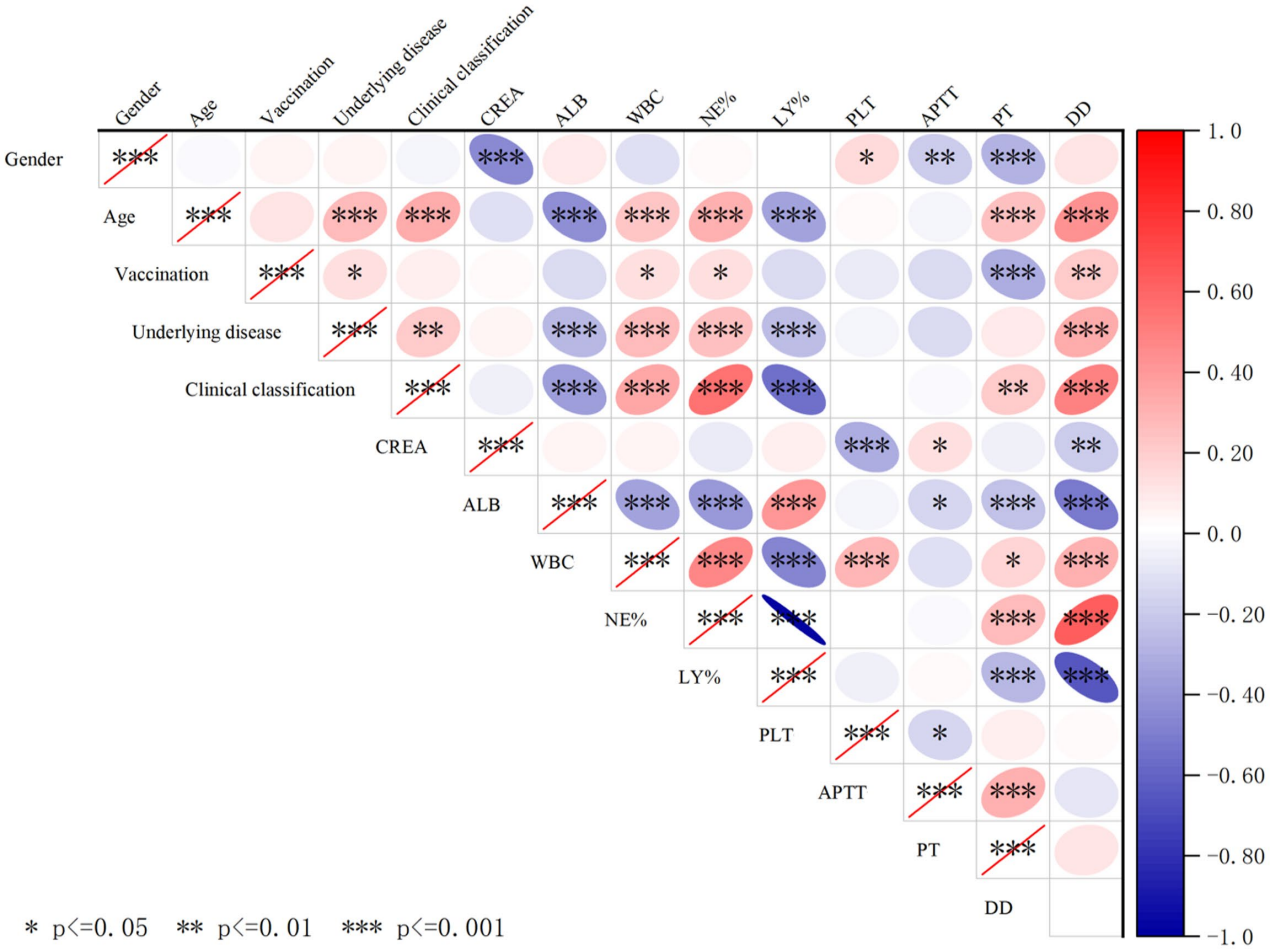
Cross-correlation analysis of demographic factors, clinical characteristics, and hematological/biochemical parameters in COVID-19 patients. The red ellipse indicates a positive correlation between the two variables, the blue ellipse indicates a negative correlation between the two variables, a darker color indicates a stronger correlation, ^*^indicates *p* < 0.05, indicates ^**^p < 0.01, and ^***^indicates *p* < 0.001, and the correlation between the two variables is considered statistically significant. Especially, clinical classification were negatively correlated with ALB and LY% and positively correlated with WBC, NE%, D-D, and underlying disease.

In this study, when patients with all three variants were combined for analysis, Pearson correlation analysis revealed that the number of days since infection was positively correlated with ALB, NE%, and PT, and negatively correlated with LY% (*p* < 0.01) (Table 5). Additionally, correlation analysis of patient sex, age, underlying diseases, vaccination status, clinical classification, and blood test parameters showed that age, underlying comorbidities, and COVID-19 clinical classification were negatively correlated with ALB and LY%, but positively correlated with WBC, NE%, and D-D. Furthermore, age was positively correlated with COVID-19 clinical classification and underlying comorbidities. The correlations between other indicators are shown in Figure 5.

**Table 5:** Correlation between blood test parameters and days of infection.

	Pearson	*p*
CREA	−0.067	0.031
ALB	0.142	<0.01
WBC	−0.034	0.196
NE	0.179	<0.01
LY	−0.183	<0.01
PLT	0.033	0.218
APTT	−0.009	0.84
PT	0.208	<0.01
DD	−0.034	0.512

The table describes the correlation between the above blood test indicators and the days of infection, *p* < 0.01 considered that the difference was statistically significant, the negative value of the Pearson value indicates that the indicator was negatively correlated with the days of infection, and the positive value of the Pearson value indicates that the indicator was positively correlated with the days of infection.

## Discussion

This study provides a comprehensive comparison of clinical and laboratory characteristics among patients infected with the B.1, BA.2, and BA.5 SARS-CoV-2 variants in Jilin Province from 2021 to 2023. Our key findings highlight distinct patterns in viral kinetics, clinical severity, and laboratory markers across variants, while also underscoring limitations that warrant consideration.

### Clinical and demographic differences

The demographic and clinical profiles of patients varied significantly between variants, likely influenced by evolving public health policies. During the BA.5 wave (December 2022–February 2023), quarantine measures were lifted, leading to broader community transmission. This shift may explain the higher proportion of elderly and comorbid patients hospitalized with BA.5, consistent with reports that Omicron BA.5 causes milder disease in younger, healthier individuals but poses greater risks to older populations with underlying conditions (Yang et al., 2022; Li et al., 2020). Logistic regression confirmed that age >60 years was strongly associated with severe disease (OR: 5.919), aligning with global trends (Qassim et al., 2022).

### Viral kinetics: Ct values and nucleic acid clearance

Viral load dynamics, assessed via ORF1ab and N gene Ct values, revealed three key observations: (1) Temporal trends: Ct values plateaued early (Day 2 post-infection) before gradually rising, with BA.2 and BA.5 showing higher variability during recovery, possibly due to sampling limitations (Zhou et al., 2022). (2) Gene-specific differences: The N gene had lower Ct values than ORF1ab in B.1 infections, supporting its higher sensitivity in diagnostics (Zhuang et al., 2021). (3) Variant-specific viral load: While BA.5 and B.1 had comparable median Ct values, BA.2 exhibited significantly lower viral loads and shorter nucleic acid clearance times. Notably, BA.5 demonstrated enhanced replicative fitness over BA.2 (Ong et al., 2023), though vaccination status may confound these findings (see limitations).

The evaluation of factors influencing the negative conversion cycle among the three variants revealed that significant differences in the time to negative conversion were only observed in patients aged ≥60 years and unvaccinated status. In contrast, the presence of underlying diseases and disease severity showed no significant impact on the negative conversion cycle. This observation may be attributed to the following factors: (1) Variant-specific pathological mechanisms: Emerging evidence suggests that Omicron variants (BA.2/BA.5) may exhibit shorter tissue persistence compared to the ancestral strain, potentially reducing clearance differences associated with disease severity (Yang et al., 2023). (2) Study population characteristics: Our hospitalized cohort may represent a narrower spectrum of disease severity than the general COVID-19 population. (3) Treatment homogeneity: The standardized antiviral treatment protocol implemented in our hospital may have minimized clearance variations related to disease severity. (4) Sample size limitations: Subgroup analyses were powered at 80% to detect differences of ≥3 days. (5) Biological plausibility: Recent studies have also indicated that while vaccination accelerates viral clearance, disease severity may not significantly prolong viral shedding time in hospitalized patients during the Omicron era (Yang et al., 2023).

### Laboratory markers: inflammation, coagulation, and organ injury

#### Inflammatory response

Elevated NE%, WBC, and D-dimer, alongside reduced LY%, correlated with disease severity, reflecting heightened inflammation in advanced COVID-19 (Zhang et al., 2020). Lymphopenia in Omicron patients may signal severe disease or age-related immune decline.

#### Renal impairment

BA.2 and BA.5 patients exhibited elevated creatinine (CREA), suggesting greater renal injury than B.1, independent of age or comorbidities. This aligns with evidence of Omicron’s tropism for renal tissue (Figure 5).

#### Coagulopathy

Thrombocytopenia and prolonged APTT/PT in severe cases implied endothelial injury and consumptive coagulopathy (Xu et al., 2020). Omicron variants (BA.2/BA.5) showed lower PLT than B.1, potentially worsening prognosis.

## Limitations and future directions

Our study has several limitations that warrant discussion. First, data gaps such as missing vaccination records (e.g., 27 BA.5 cases) and uneven vaccine rollout (none in the B.1 cohort) limited the assessment of vaccine efficacy. Second, subgroup heterogeneity was not fully addressed; simplified vaccinated/unvaccinated comparisons failed to account for dosing intervals and waning immunity, highlighting the need for larger, stratified studies (Kandel et al., 2023; Tozer et al., 2022). Third, unmeasured confounders, including co-infections (e.g., influenza/RSV) that may alter viral kinetics, were not systematically evaluated (Contes and Liu, 2025). Future research should prioritize these aspects to strengthen the robustness of conclusions.

## Conclusion

While Omicron variants (BA.2/BA.5) may drive milder disease in younger populations, they pose significant risks to older, comorbid individuals, with distinct impacts on viral persistence, organ injury, and coagulation. Our findings underscore the need for variant-specific clinical monitoring and highlight critical gaps—particularly in vaccine effectiveness and co-infection dynamics—for future research.

## Data Availability

The raw data supporting the conclusions of this article will be made available by the authors, without undue reservation.

## References

[ref1] Alkhatib M. Svicher V. Salpini R. Ambrosio F. A. Bellocchi M. C. Carioti L.. (2021). SARS-CoV-2 variants and their relevant mutational profiles: update summer 2021. Microbiol. Spectr. 9:e0109621. doi: 10.1128/Spectrum.01096-21, PMID: 34787497 PMC8597642

[ref2] Arachchillage D. R. J. Laffan M. (2020). Abnormal coagulation parameters are associated with poor prognosis in patients with novel coronavirus pneumonia. J. Thromb. Haemost. 18, 1233–1234. doi: 10.1111/jth.14820, PMID: 32291954 PMC7262191

[ref3] Aranha C. Patel V. Bhor V. Gogoi D. (2021). Cycle threshold values in RT-PCR to determine dynamics of SARS-CoV-2 viral load: an approach to reduce the isolation period for COVID-19 patients. J. Med. Virol. 93, 6794–6797. doi: 10.1002/jmv.27206 34264527 PMC8426941

[ref4] Cheng Y. Luo R. Wang K. Zhang M. Wang Z. Dong L.. (2020). Kidney disease is associated with in-hospital death of patients with COVID-19. Kidney Int. 97, 829–838. doi: 10.1016/j.kint.2020.03.005, PMID: 32247631 PMC7110296

[ref5] Colson P. Lavagna C. Delerce J. Groshenry G. Yahi N. Fantini J.. (2022). First detection of the SARS-CoV-2 Omicron BA.5/22B in Monaco. Microorganisms 10:1952. doi: 10.3390/microorganisms10101952, PMID: 36296228 PMC9607325

[ref6] Contes K. M. Liu B. M. (2025). Epidemiology, clinical significance, and diagnosis of respiratory viruses and their co-infections in the post-COVID era. Pathogens 14:262. doi: 10.3390/pathogens14030262, PMID: 40137747 PMC11944763

[ref7] Flisiak R. Rzymski P. Zarębska-Michaluk D. Ciechanowski P. Dobrowolska K. Rogalska M.. (2023). Variability in the clinical course of COVID-19 in a retrospective analysis of a large real-world database. Viruses 15:149. doi: 10.3390/v15010149, PMID: 36680188 PMC9863894

[ref8] Gao W. Li Z. Guan Q. Cui W. Zheng B. Ding Q.. (2023). Characterization and analysis of linear epitopes corresponding to SARS-CoV-2 outbreak in Jilin Province, China. J. Med. Virol. 95:e28323. doi: 10.1002/jmv.28323, PMID: 36401153

[ref9] Huang C. Wang Y. Li X. Ren L. Zhao J. Hu Y.. (2020). Clinical features of patients infected with 2019 novel coronavirus in Wuhan, China. Lancet 395, 497–506. doi: 10.1016/s0140-6736(20)30183-5 31986264 PMC7159299

[ref10] Kandel C. E. Banete A. Taylor M. Llanes A. McCready J. Crowl G.. (2023). Similar duration of viral shedding of the severe acute respiratory coronavirus virus 2 (SARS-CoV-2) delta variant between vaccinated and incompletely vaccinated individuals. Infect. Control Hosp. Epidemiol. 44, 1002–1004. doi: 10.1017/ice.2022.124, PMID: 35598890 PMC9237490

[ref11] Levi M. Thachil J. Iba T. Levy J. H. (2020). Coagulation abnormalities and thrombosis in patients with COVID-19. Lancet Haematol. 7, e438–e440. doi: 10.1016/s2352-3026(20)30145-9, PMID: 32407672 PMC7213964

[ref12] Li K. Wu J. Wu F. Guo D. Chen L. Fang Z.. (2020). The clinical and chest CT features associated with severe and critical COVID-19 pneumonia. Investig. Radiol. 55, 327–331. doi: 10.1097/rli.0000000000000672, PMID: 32118615 PMC7147273

[ref13] Lippi G. Plebani M. Henry B. M. (2020). Thrombocytopenia is associated with severe coronavirus disease 2019 (COVID-19) infections: a meta-analysis. Clin. Chim. Acta 506, 145–148. doi: 10.1016/j.cca.2020.03.022, PMID: 32178975 PMC7102663

[ref14] Liu B. M. Yao Q. Cruz-Cosme R. Yarbrough C. Draper K. Suslovic W.. (2025). Genetic conservation and diversity of SARS-CoV-2 envelope gene across variants of concern. J. Med. Virol. 97:e70136. doi: 10.1002/jmv.70136, PMID: 39744807 PMC12228529

[ref15] Long B. Carius B. M. Chavez S. Liang S. Y. Brady W. J. Koyfman A.. (2022). Clinical update on COVID-19 for the emergency clinician: presentation and evaluation. Am. J. Emerg. Med. 54, 46–57. doi: 10.1016/j.ajem.2022.01.028, PMID: 35121478 PMC8779861

[ref16] Miranda R. L. Guterres A. de Azeredo Lima C. H. Filho P. N. Gadelha M. R. (2021). Misinterpretation of viral load in COVID-19 clinical outcomes. Virus Res. 296:198340. doi: 10.1016/j.virusres.2021.198340, PMID: 33592214 PMC7881726

[ref17] Ong C. P. Ye Z. W. Tang K. Liang R. Xie Y. Zhang H.. (2023). Comparative analysis of SARS-CoV-2 Omicron BA.2.12.1 and BA.5.2 variants. J. Med. Virol. 95:e28326. doi: 10.1002/jmv.28326 36411262

[ref18] Piersiala K. Kakabas L. Bruckova A. Starkhammar M. Cardell L. O. (2022). Acute odynophagia: a new symptom of COVID-19 during the SARS-CoV-2 Omicron variant wave in Sweden. J. Intern. Med. 292, 154–161. doi: 10.1111/joim.13470, PMID: 35170099 PMC9115132

[ref19] Qassim S. H. Hasan M. R. Tang P. Chemaitelly H. Ayoub H. H. Yassine H. M.. (2022). Effects of SARS-CoV-2 Alpha, Beta, and Delta variants, age, vaccination, and prior infection on infectiousness of SARS-CoV-2 infections. Front. Immunol. 13:984784. doi: 10.3389/fimmu.2022.984784, PMID: 36177014 PMC9513583

[ref20] Sohn Y. J. Shin P. J. Oh W. S. Kim E. Kim Y. Kim Y. K. (2022). Clinical characteristics of patients who contracted the SARS-CoV-2 Omicron variant from an outbreak in a single hospital. Yonsei Med. J. 63, 790–793. doi: 10.3349/ymj.2022.63.8.790, PMID: 35914762 PMC9344273

[ref21] Terpos E. Ntanasis-Stathopoulos I. Elalamy I. Kastritis E. Sergentanis T. N. Politou M.. (2020). Hematological findings and complications of COVID-19. Am. J. Hematol. 95, 834–847. doi: 10.1002/ajh.25829, PMID: 32282949 PMC7262337

[ref22] Tozer K. Sjaarda C. P. Moslinger E. Wong H. Mubareka S. Maguire F.. (2022). Comparison of SARS-CoV-2 viral loads in the nasal mucosa of patients infected with BA.1, BA.2, or BA.5 Omicron lineages. Open Forum Infect. Dis. 9:ofac564. doi: 10.1093/ofid/ofac564, PMID: 36483184 PMC9620335

[ref23] Wei P.-F. (2020). Diagnosis and treatment protocol for novel coronavirus pneumonia (trial version 7). Chin. Med. J. 133, 1087–1095. doi: 10.1097/cm9.0000000000000819, PMID: 32358325 PMC7213636

[ref24] Xu P. Zhou Q. Xu J. (2020). Mechanism of thrombocytopenia in COVID-19 patients. Ann. Hematol. 99, 1205–1208. doi: 10.1007/s00277-020-04019-0, PMID: 32296910 PMC7156897

[ref25] Yang Y. Guo L. Yuan J. Xu Z. Gu Y. Zhang J.. (2023). Viral and antibody dynamics of acute infection with SARS-CoV-2 Omicron variant (B.1.1.529): a prospective cohort study from Shenzhen, China. Lancet Microbe 4, e632–e641. doi: 10.1016/s2666-5247(23)00139-8, PMID: 37459867

[ref26] Yang W. Yang S. Wang L. Zhou Y. Xin Y. Li H.. (2022). Clinical characteristics of 310 SARS-CoV-2 Omicron variant patients and comparison with Delta and Beta variant patients in China. Virol. Sin. 37, 704–715. doi: 10.1016/j.virs.2022.07.014, PMID: 35948265 PMC9357284

[ref27] Zhang J. J. Dong X. Cao Y. Y. Yuan Y. D. Yang Y. B. Yan Y. Q.. (2020). Clinical characteristics of 140 patients infected with SARS-CoV-2 in Wuhan, China. Allergy 75, 1730–1741. doi: 10.1111/all.14238, PMID: 32077115

[ref28] Zhou K. Hu B. Zhao X. Chi H. Pan J. Zheng Y.. (2022). Longitudinal observation of viral load in patients infected with Omicron variant and its relationship with clinical symptoms. Front. Microbiol. 13:1037733. doi: 10.3389/fmicb.2022.1037733, PMID: 36713203 PMC9880150

[ref29] Zhuang Q. Z. Li Z. Z. Chao Y. Li F. Ge Y. Y. Sha Y. H.. (2021). Comparative performance of four nucleic acid amplification tests for SARS-CoV-2 virus. Clin. Lab. 67. doi: 10.7754/Clin.Lab.2020.201025 34258963

